# Local Polynomial Estimation of Heteroscedasticity in a Multivariate Linear Regression Model and Its Applications in Economics

**DOI:** 10.1371/journal.pone.0043719

**Published:** 2012-09-17

**Authors:** Liyun Su, Yanyong Zhao, Tianshun Yan, Fenglan Li

**Affiliations:** 1 School of Mathematics and Statistics, Chongqing University of Technology, Chongqing, China; 2 Library, Chongqing University of Technology, Chongqing, China; King Abdullah University of Science and Technology, Saudi Arabia

## Abstract

Multivariate local polynomial fitting is applied to the multivariate linear heteroscedastic regression model. Firstly, the local polynomial fitting is applied to estimate heteroscedastic function, then the coefficients of regression model are obtained by using generalized least squares method. One noteworthy feature of our approach is that we avoid the testing for heteroscedasticity by improving the traditional two-stage method. Due to non-parametric technique of local polynomial estimation, it is unnecessary to know the form of heteroscedastic function. Therefore, we can improve the estimation precision, when the heteroscedastic function is unknown. Furthermore, we verify that the regression coefficients is asymptotic normal based on numerical simulations and normal Q-Q plots of residuals. Finally, the simulation results and the local polynomial estimation of real data indicate that our approach is surely effective in finite-sample situations.

## Introduction

The heteroscedasticity in classical linear regression model is defined by the variances of random items and which are not the same for different explanatory variables and observations [1], [2]. Especially the heteroscedasticity is usually inevitable when we study the cross-sectional data [3]–[6]. When there is a heteroscedasticity in a linear regression model, estimations of parameters we obtained by ordinary least squares estimation (OLS) are still linear and unbiased [7]–[10]. However, the efficiency is bad [11], [12]. This could lead to a uncorrect statistical diagnosis for the parameters’ significance test. Similarly, it unnecessarily enlarges the confidence interval when we estimate the parameter interval. Besides, the accuracy of predictive value may lower when we estimate with the regression model that we obtained. In order to solve the problem above, we can use generalized least squares estimation (GLS) when the covariance matrix of the random items is known. If it is unknown, we usually use two-stage least squares estimate. In other words, we first estimate variances of the residual error, and then the generalized least squares estimator is used to obtain the coefficients of the model by using the estimate of variances of the random items. However, the traditional estimation method is that we suppose the residual error variances as a certain parametric model. So estimations we obtained are always inaccurate. For a heteroscedasticity model which has only one explanatory variable, we have discussed in detail and given a rigorous proof in [13]. In this paper, we try to apply multivariate local polynomial fitting to random item variances as the first step, and then generalized least squares estimation is used to estimate the coefficients of the model. On the one hand, because of local polynomial fitting’s various nice statistical properties, the estimations obtained with this technology also have the same good statistical properties [14]. On the other hand, we exploit a heteroscedastic regression model rather than the artificial structure of heteroscedasticity. Then, we can directly get the heteroscedastic function based on the nonparametric technique, which shows the relationship between variance function of random items and explanatory variables from regression results [15]. Thus, it is unnecessary to test heteroscedasticity of the model. Particularly, the estimated value by multivariate local polynomial fitting are more accurate than that by the traditional method and univariate local polynomial fitting.

The rest of this paper is organized as follows. The multivariate local polynomial estimation adopted is explained in detail in Section Methods [16]–[18]: in its the first Subsection we study multivariate estimation with local polynomial fitting. The second subsection contains selections of parameters. Estimations of model coefficients and heteroscedastic function are presented in the third subsection Parametric estimations with multivariate local polynomial regression. In Sections Results and Discussion, we firstly give two models, do the simulations, then collect some real data, and use the local polynomial estimating the coefficients, respectively. Finally, Section Conclusions conclude.

## Methods

Multivariate local polynomial fitting is an attractive method both from theoretical and practical point of view. It possesses a smaller mean squared error than that of classical kernel Nadaraya-Watson estimator which leads to an undesirable form of the bias and the Gasser-Muller estimator which has to pay a price in variance when dealing with a random design model. It also possesses other advantages [19], [20]. In this Section, we briefly outline the idea of the extension of multivariate local polynomial fitting to multivariate linear regression.

### Multivariate Estimator with Local Polynomial Fitting

We treat the m-dimension estimation problem where the measured data 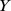 at the position 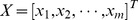 is given by
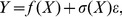
(1)where 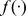 is the regression function to be estimate, 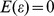, 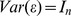 (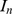 denoting the identity matrix of order 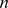), and 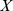 and 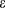 are independent. We always denote the conditional variance of 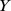 given 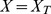 by 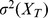 and the marginal density of 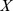, that is, the design density, by 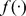.

While the specific form of 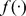 may remain unspecific, if we assume that the 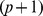 th derivative of 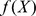 at the point 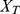 exsits, then in order to estimate the value at this point, we can rely on a generic local expansion of the function about this point. Specifically, for 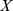 in a neighborhood of 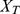, a 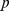-term Taylor expansion gives,
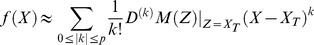
(2)where



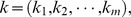


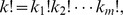


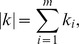


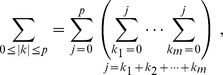


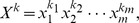


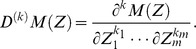



Given the series 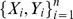, this polynomial is fitted locally by a weighted least squares regression problem: minimize.
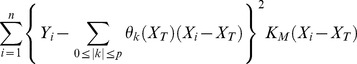
(3)where 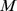 is a bandwidth matrix controlling the size of the local neighborhood and 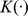 is the kernel function which penalizes both geometric and radiometric distances, where 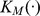 is defined as [21]



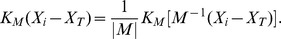



Denote by 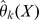 the solution to the least squares problem (3)). It is clear from the Taylor expansion in (2) that 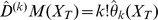 is an estimator for 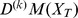.

It is more convenient to work with matrix notation. For the weighted least squared problem, a matrix form can be depicted by
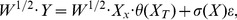
(4)where



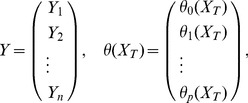
(5)

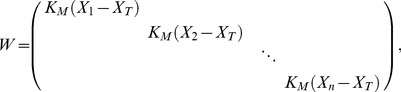
(6)and



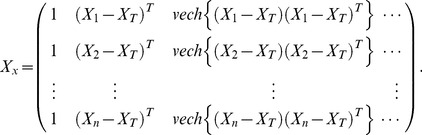
(7)We then have the least squared solution with multivariate local polynomial fitting [22].
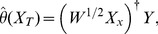
(8)where 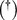 denotes pseudo-inverse, or when 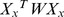 is inverse, the estimation can be written by



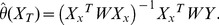
(9)Then, we can get the estimation 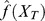,
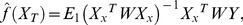
(10)where 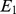 is a column vector (the same size of a in (5)) with the first element equal to 1, and the rest equal to zero, that is, 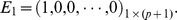


Computing the 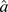 will suffer from large computational cost. We can use the recursive least squared method to reduce the computation complexity, and it is very powerful especially in the local polynomial fitting problems. There are several important issues about the bandwidth, the order of multivariate local polynomial function and the kernel function which have to be discussed. The three problems will be presented in following subsection.

### Parameters Selections

For the multivariate local polynomial estimator, there are three important problems which have significant influence to the estimation accuracy and computational complexity [23].

First of all, there is the choice of the bandwidth matrix, which plays a rather crucial role. The bandwidth matrix 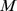 is taken to be a diagonal matrix. For simplification, the bandwidth matrix is designed into 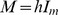. Therefore, the most important thing is to find the bandwidth 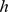 [24], [25]. In theory, there exists a optimal bandwidth 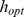 in the meaning of mean integrated square error(MISE), fulfilling the equality
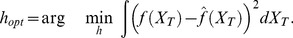
(11)


However, the theoretical bandwidth 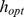 in formula (23) can not be directly calculated. Here, we propose a search method to select the bandwidth: Compare values of the objective function as the bandwidth 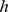 from small to large, and then find out the optimal bandwidth which minimize the objective function.

Suppose that 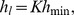 where 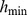 is the minimum, 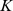 is coefficient of expansion. We search a bandwidth 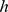 to minimize the objective function in the interval 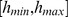, where the objective function refers to the prediction mean square error (MSE), denoted by 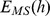.

Firstly, we assume 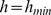, then increase 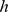 by efficient of expansion 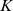 and calculate value of objective function for each 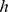. Stop down when 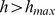, and choose a bandwidth 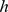 which minimizes 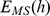 as the approximate optimal bandwidth. 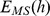 can be taken place by a estimation
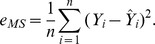



In this paper, we choose
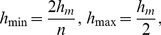
(12)where 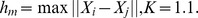 Compared with other methods, this method is more convenient.

In order to closer to the ideal optimal bandwidth, we search once again by narrowing the interval on the basis of the above searching process. Supposing 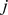 is the bandwidth which make 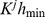 optimal in the above searching process. Now, divide the small interval 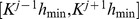 into 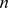 equal intervals. Supposing
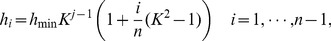
(13)among these 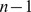 bandwidths, the approximate optimal bandwidth is the one that makes 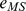 minimize.Obviously, this search method can quickly select the right bandwidth.

Another issue in multivariate local polynomial fitting is the choice of the order of the polynomial. For a given bandwidth 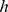, a large value of 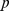 would expectedly reduce the modeling bias, but would cause a large variance and a considerable computational cost. Since the bandwidth is used to control the modeling complexity, and due to the sparsity of local data in multi-dimensional space, a higher-order polynomial is rarely used. So we apply the local quadratic regression to fit the model (that is to say, 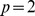).

The third issue is the selection of the kernel function. In this paper, we choose the spherical 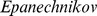 kernel as kernel function
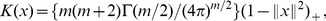
(14)where 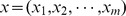, 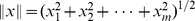, and 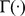 represents 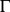 function. This is the optimal kernel function, see [14], [19], [20].

### Parametric Estimations with Multivariate Local Polynomial Regression

Let the dependent variable 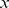 and the explanatory variable 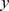 fulfill the following regression model £°.
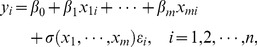
(15)where 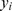 are the observations and 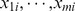 are independent variables. Denote
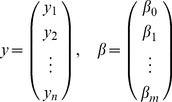
(16)and



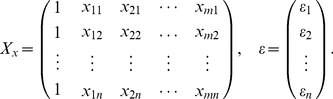
(17)Therefore, the equation (15) can be abbreviated as
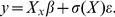
(18)


Suppose that

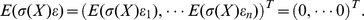

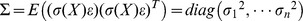
,

where 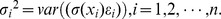

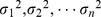
 are all not equal, that is, there is a heteroscedasticity in model (18). Therefore, the WLS of 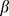 is.
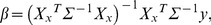
(19)or it can be written as
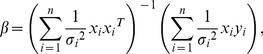
(20)where 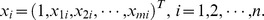 If covariance matrix 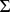 of 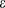 is known, formula (20) can be estimated. Equation (20) is considered as the weighted least squares estimate of 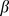 and it possesses nice qualities. However, in many practical situations, the form of 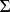 is unknown. Therefore, the so-called two-stage method of estimation is used to solve the heteroscedasticity problem. It can be depicted as follows: first, apply multivariate local polynomial fitting to get the estimate 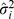 of 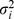, and then we can obtain the estimate 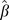 of 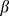 by using equation (20). The estimator follows that

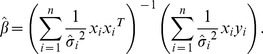
(21)Because of 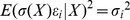, we construct the following regression model in order to estimate 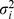,
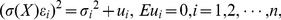
(22)where 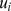 is the difference between 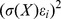 and its expectation. Suppose that 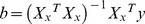 is the OLS of model (18). Although the ordinary least squares estimate 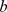 is ineffective, it is still consistent. Therefore, the corresponding residuals hold that



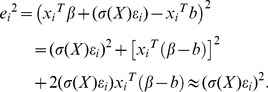
(23)Consequently, we can approximately get
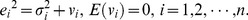
(24)


It can be taken as a regression model, in which the variance function is regression function and the squared residuals 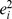 are dependent variables. In order to estimate this model, parameter estimation method would usually be taken in some articles. In other words, they suppose 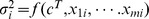, where the form of 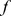 is known and 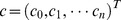 are the parameters to be estimated. Note that what we usually discuss about more and more detail are 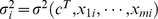, 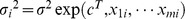 and so on [26]. However, the discussion of these models requires that the analyst have a better understanding for the background in practical problems. As an example, variance of corporate profits is often in direct proportion with family income. Since the variance function must be non-negative, a non-parametric method is proposed to fit 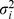. This method can be depicted as follows. Then, the 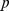-order local polynomial estimate of the variances function 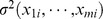 is according to formula (2). Using the least squares method for the data around the local window, we can estimate the local intercept via minimizing
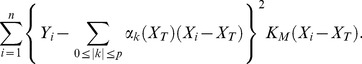
(25)


Furthermore, the solution vector can be written as
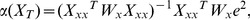
(26)where the weighted matrix



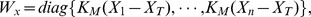
the the design matrix
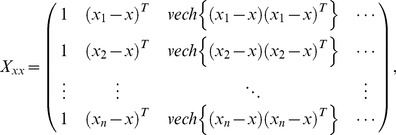
(27)and 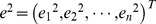. Consequently, the estimated variance function is 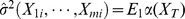. Finally, we can get two-stage estimate 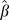 of 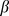 by substituting estimate 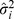 of 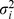 into equation (21).

## Results and Discussion

### Simulations and Analysis

In this section, we give the following model to discuss the qualities of 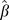 under the limited sample. Considering the practical background which is applied to economics, we assume the variance function of the following two forms.

#### Model 1

Denote the linear model by
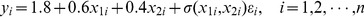
(28)where 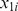 and 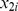 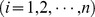 are independent variables. Here, we assume the variance function of the error term 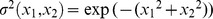.

**Figure 1:**
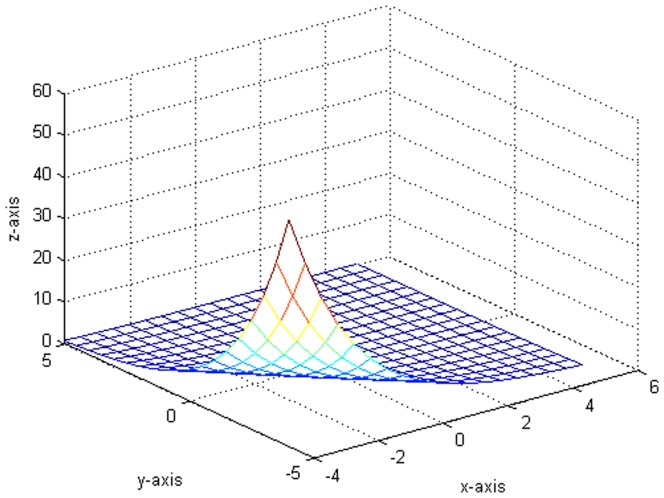
The fitted plot after 10000 replicates.

Step 1: Firstly, obtain the estimation of two coefficients in model (28) with the ordinary least squares estimation. Second, calculate the squares of the residuals 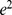. Third, do the local polynomial regression on the model (24) Figure 1 is the result after 10000 replicates, where 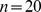, 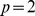, and the kernel function is given by.
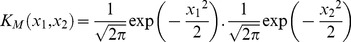
(29)


In the kernel function, the ranges of 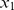 and the same with 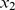 are 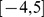, and the optimal bandwidth matrix is 
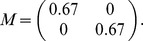
 The criteria of selecting 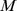 is according to MISE, which can be given by
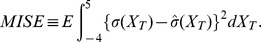



That is 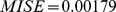.

**Figure 2:**
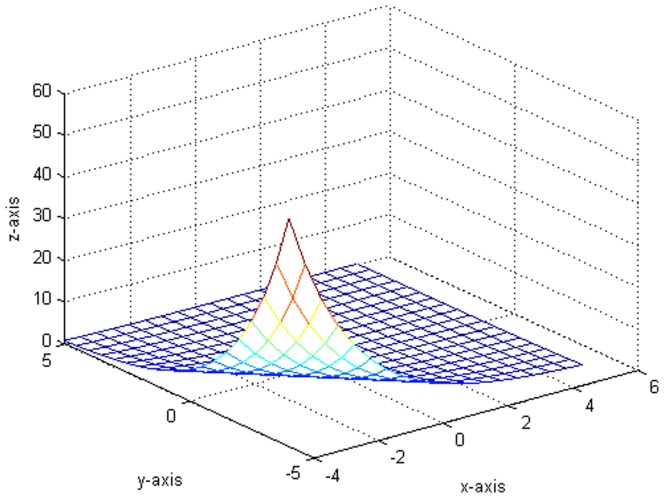
The original plot of the variance function.

Minimizing the asymptotic MISE with respect to the bandwidth parameter 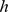 results in a bandwidth, called the asymptotically optimal bandwidth or simply optimal bandwidth. So we can obtain the optimal bandwidth matrix 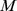. Figure 2 shows the plot which generates from the variance function.
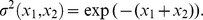



**Figure 3:**
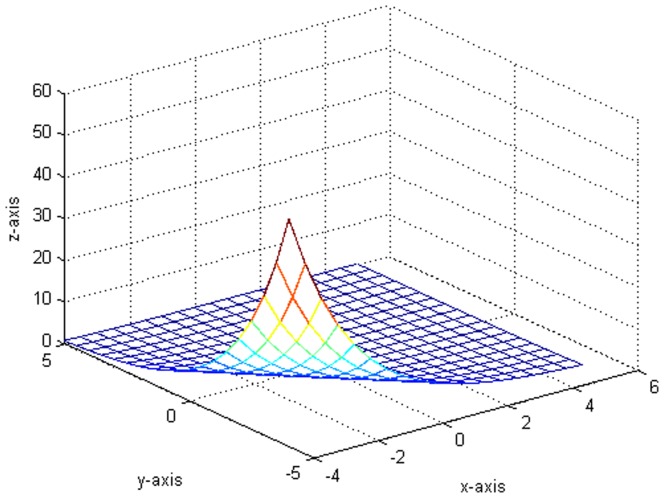
The plot of drawing fitted figure and original figure together.

Figure 3 indicates the variance of the error term function and the fitting functions drawn in the same situation. Besides, we compare some fitted value with the true value in Table 1. It is worth pointing out that our fitting is very close to the real value from Figures 1, 2, 3 and Table 1.

**Table 1:** True value and fitted value at points.

Points	True value	Fitted value
(−0.5, −0.5)	1.64870	1.6345
(−0.5,0.0)	1.28400	1.27360
(−0.5,0.5)	1.00000	0.99139
(−0.5,1.0)	0.77880	0.77245
(0.0, −0.5)	1.28400	1.27360
(0.0,0.0)	1.00000	0.99230
(0.0,0.5)	0.77880	0.77245
(0.0,1.0)	0.60653	0.60186
(0.5, −0.5)	1.00000	0.99139
(0.5,0.0)	0.77880	0.77245
(0.5,0.5)	0.60653	0.60131
(0.5,1.0)	0.47237	0.46852
(1.0, −0.5)	0.77880	0.77245
(1.0,0.0)	0.60653	0.60186
(1.0,0.5)	0.47237	0.46852
(1.0,1.0)	0.36788	0.36505

**Figure 4:**
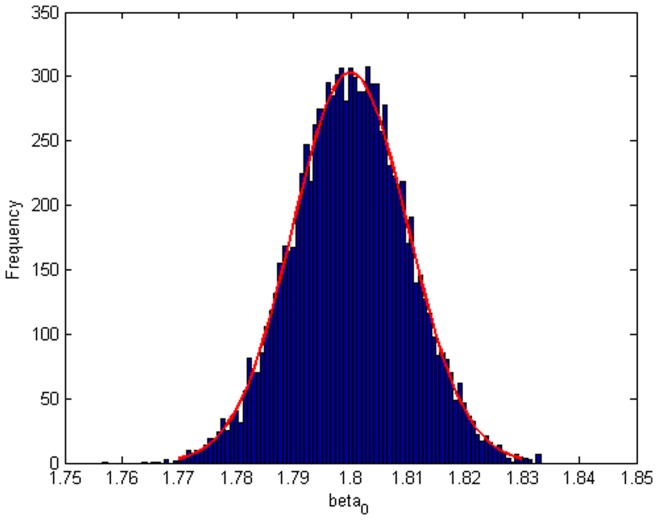
Histogram and asymptotic distribution for 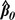 of GLS when the variance function of the error term is 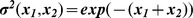.

**Figure 5:**
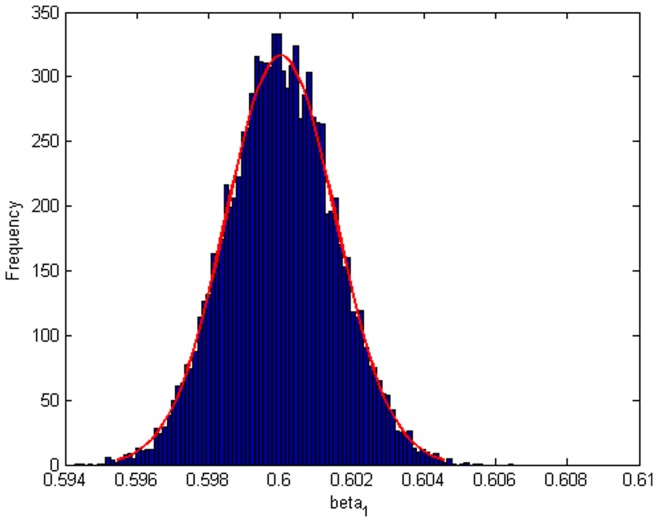
Histogram and asymptotic distribution for 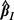 of GLS when the variance function of the error term is 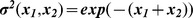.

**Figure 6:**
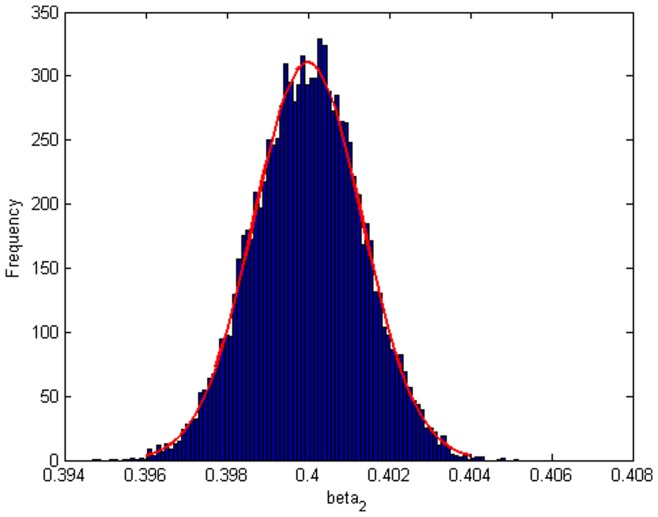
Histogram and asymptotic distribution for 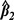 of GLS when the variance function of the error term is 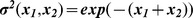.

**Figure 7:**
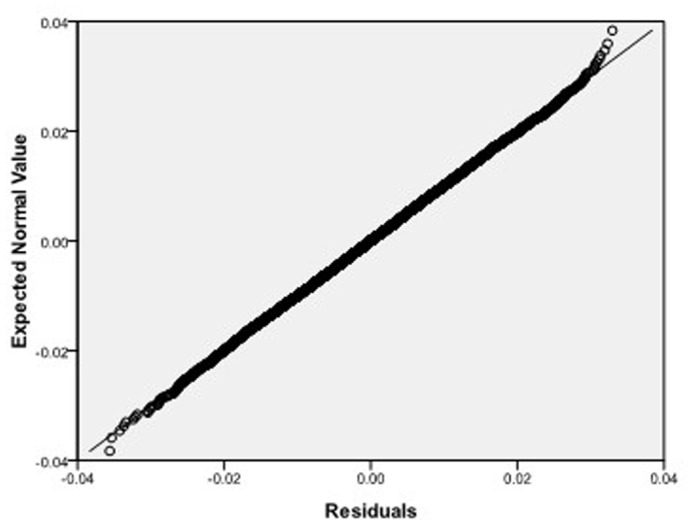
Normal Q-Q plot of residuals of 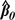 of GLS when the variance function of the error term is 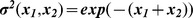.

**Figure 8:**
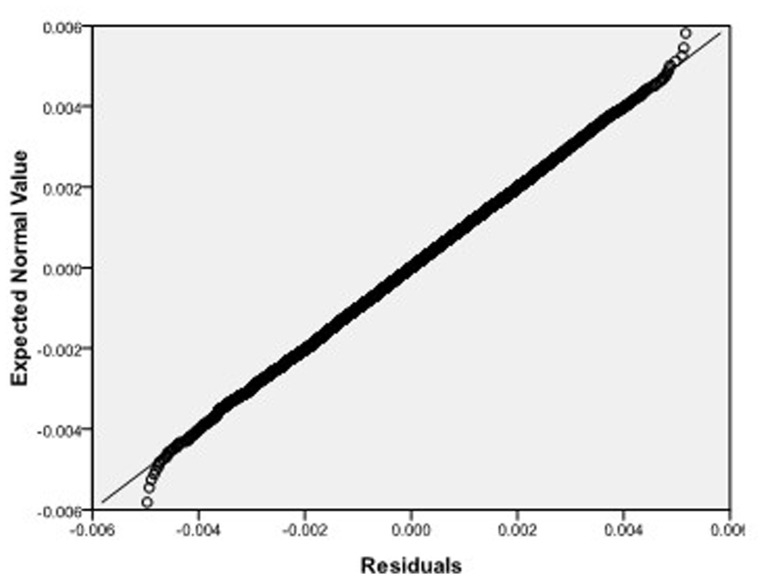
Normal Q-Q plot of residuals of 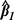 of GLS when the variance function of the error term is 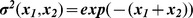.

**Figure 9:**
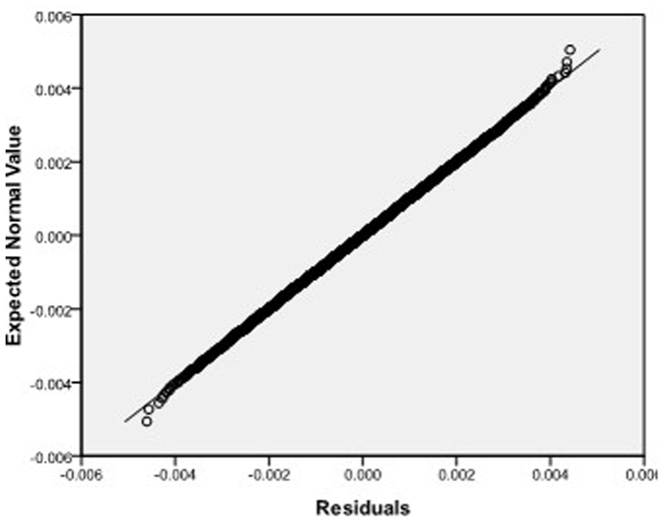
Normal Q-Q plot of residuals of 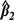 of GLS when the variance function of the error term is 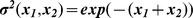.

**Figure 10:**
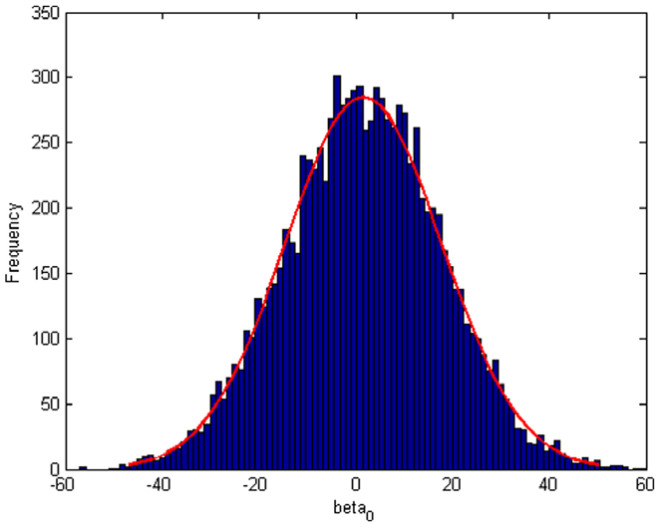
Histogram and asymptotic distribution for 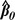 of OLS when the variance function of the error term is 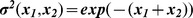.

**Figure 11:**
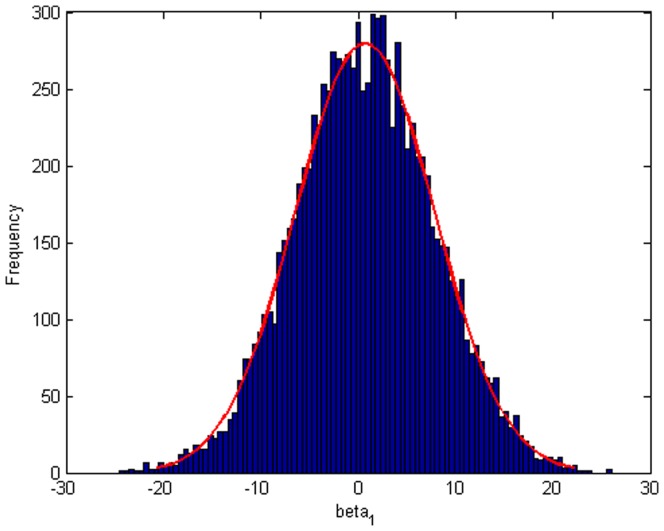
Histogram and asymptotic distribution for 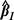 of OLS when the variance function of the error term is 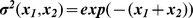.

**Figure 12:**
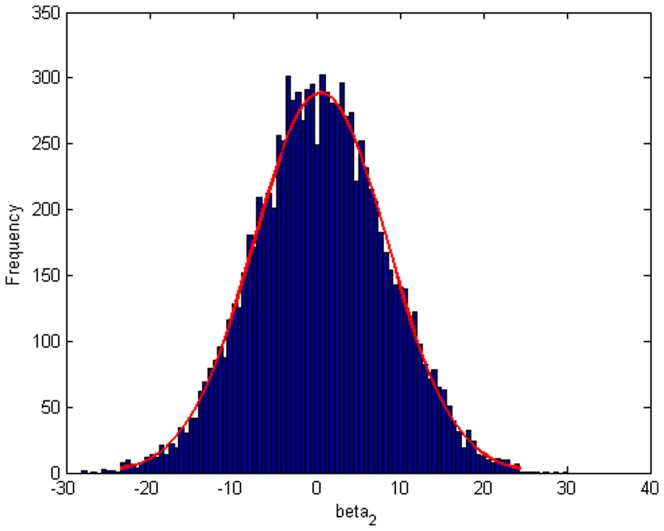
Histogram and asymptotic distribution for 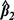 of OLS when the variance function of the error term is 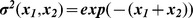.

**Figure 13:**
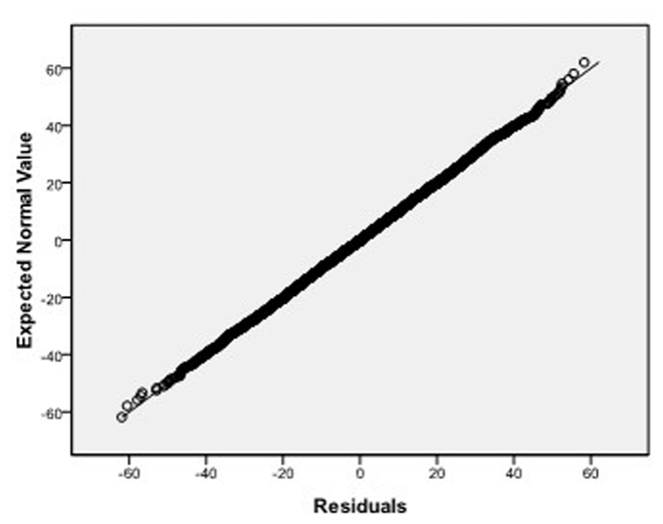
Normal Q-Q plot of residuals of 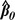 of OLS when the variance function of the error term is 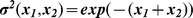.

**Figure 14:**
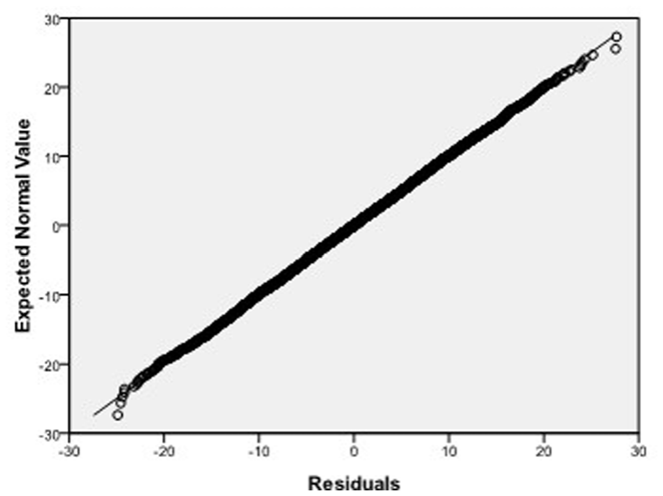
Normal Q-Q plot of residuals of 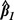 of OLS when the variance function of the error term is 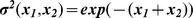.

**Figure 15:**
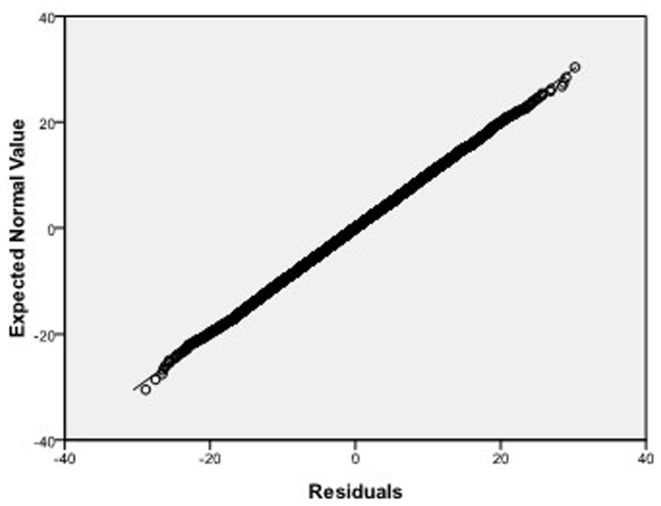
Normal Q-Q plot of residuals of 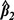 of OLS when the variance function of the error term is 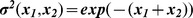.

Step 2: We substitute 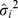 which obtained from step 1 into model (20), then we will get 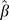. Figures 4, 5, 6 depict the histograms and asymptotic distributions of 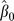, 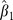, and 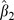, respectively, which we do 10000 replicates with GLS and choose 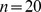. It is easy to see from Figures 4, 5, 6 that the estimated distributions of parameters subject to normal asymptotically, which the proofs are in [27]–[30]. Further, we give normal Q-Q plots of residuals of estimated parameters in Figures 7, 8, 9. As illustrated in Figures 10, 11, 12, the histograms and asymptotic distributions of 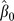, 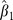, and 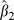 can be get by using OLS, respectively. Similarly, the normal Q-Q plots of residuals are depicted in Figures 13, 14, 15. However, the results are obviously unsatisfying in Figures 10, 11, 12, 13, 14, 15. Besides, the fitted value and true value of GLS and ones of OLS are listed in Table 2. Therefore, we can conclude that our fitting is very perfect. Here, we define the relative error as 
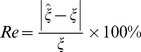
, where 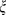 is a true value, and 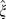 is a fitted value. So the GLS estimates of parameters are much better than OLS according to Table 2.

**Table 2:** Two-stage estimates of 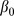, 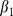, and 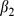 of GLS and OLS.

true value	1.8	0.6	0.4
GLS fitted value	1.79996	0.60014	0.39997
GLS relative error	0.0022%	0.233%	0.0075%
OLS fitted value	1.84603	0.57973	0.38452
OLS relative error	2.5572%	3.3783%	3.8700%

#### Model 2

**Figure 16:**
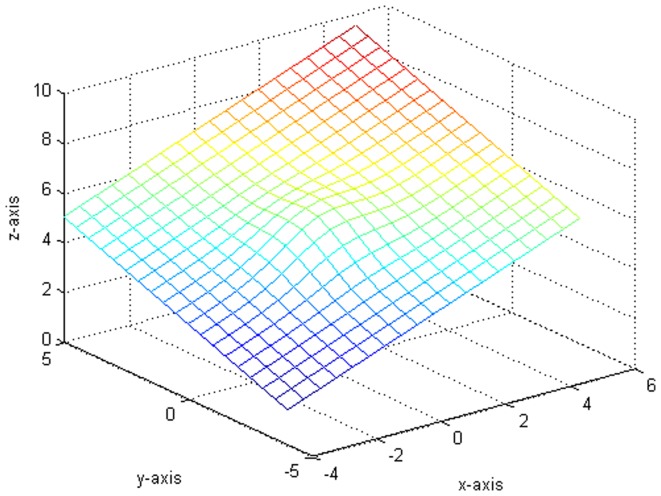
The fitted plot after 10000 replicates.

**Figure 17:**
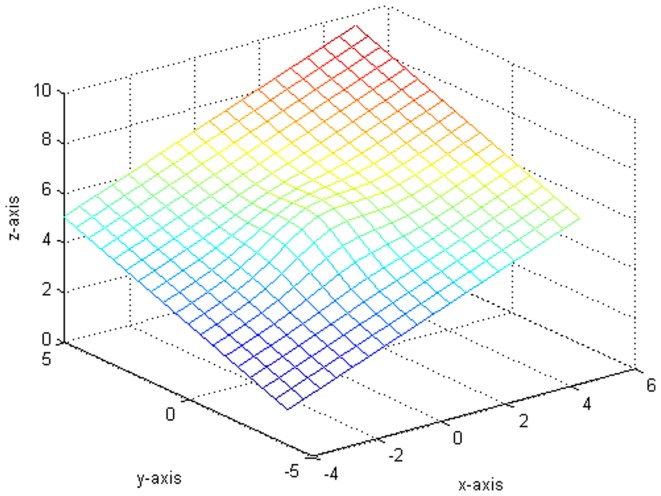
The original plot of the variance function.

In formula (28), we assume the error term function is



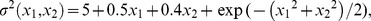
.and the optimal bandwidth matrix is 
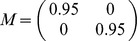
, where 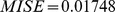. The process of parameter’s estimation and calculation is the same with Model 1. Also we choose 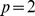, the ranges of 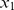 and the same with 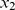 are 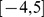. Besides, the kernel function is still defined as equation (29). We do a total of 10000 replicates. Figure 16 shows the estimated variance function 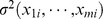, where the selection of optimal bandwidth matrix is similar with that in step 1. Figure 17 shows the plot which generates from the variance function



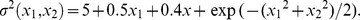



**Figure 18:**
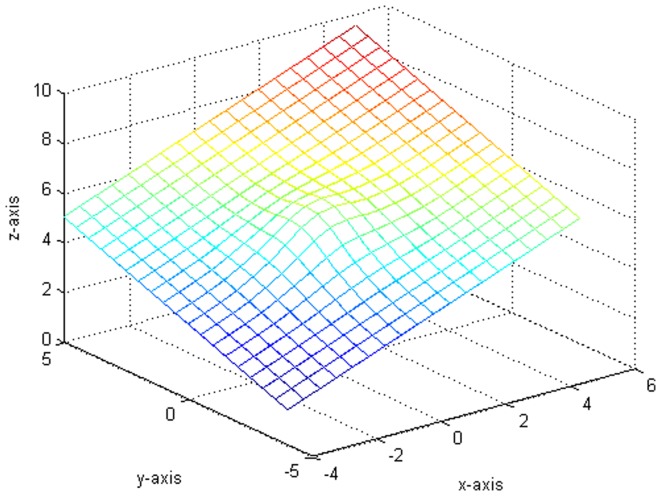
The plot of drawing fitted plot and original plot together.

**Figure 19:**
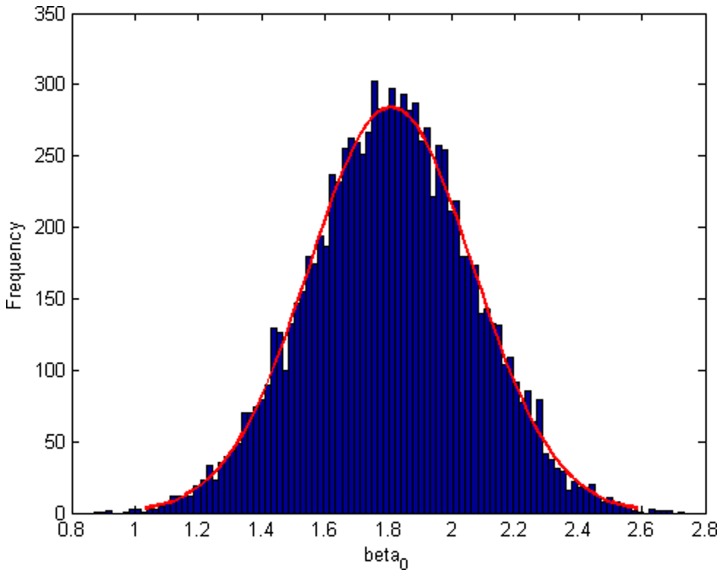
Histogram and asymptotic distribution for 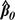 of GLS when the variance function of the error term is 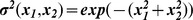.

**Figure 20:**
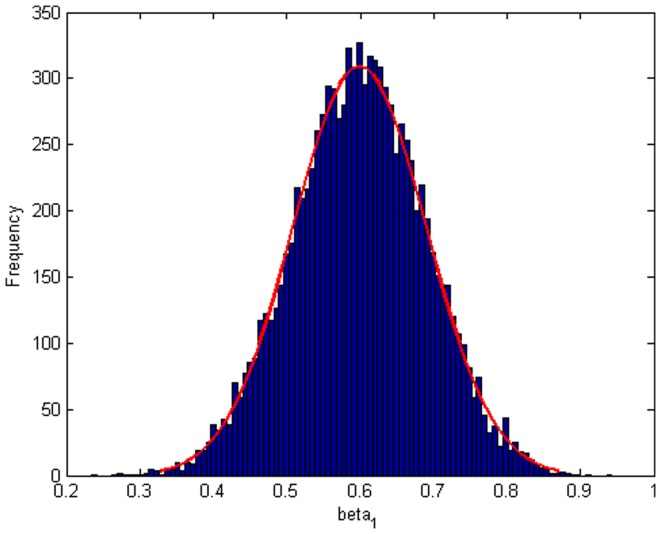
Histogram and asymptotic distribution for 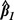 of GLS when the variance function of the error term is 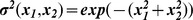.

**Figure 21:**
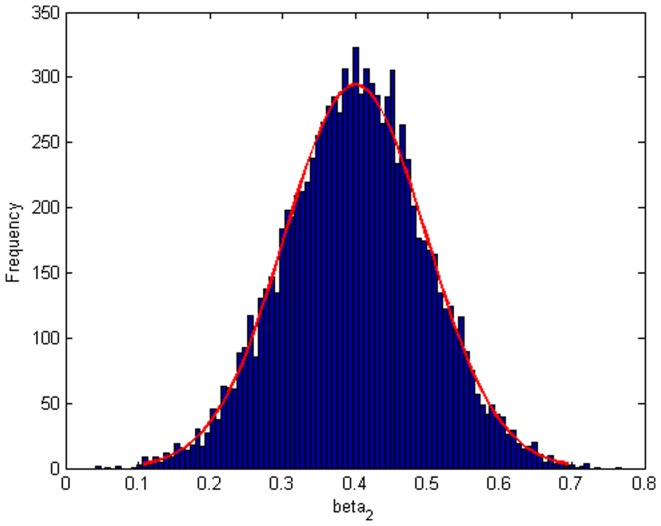
Histogram and asymptotic distribution for 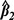 of GLS when the variance function of the error term is 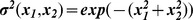.

**Figure 22:**
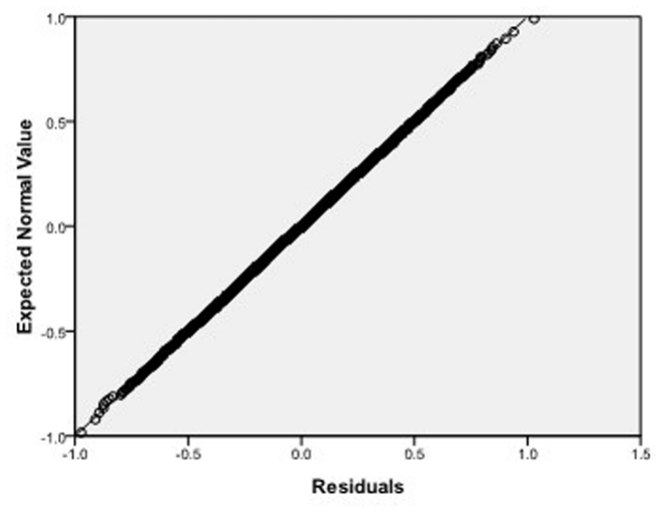
Normal Q-Q plot of residuals of 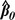 of GLS when the variance function of the error term is 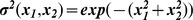.

**Figure 23:**
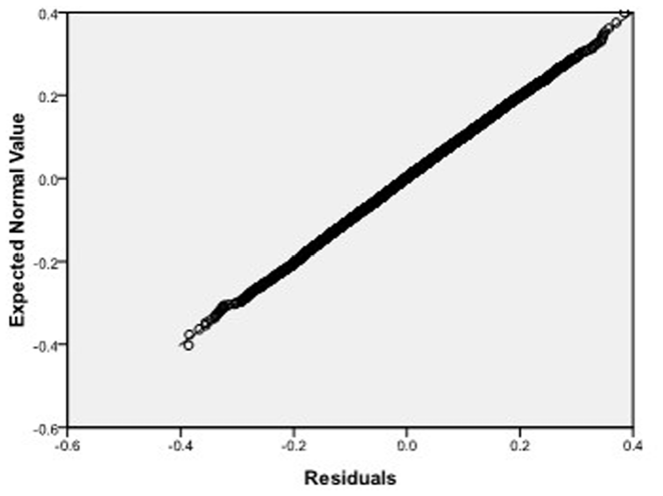
Normal Q-Q plot of residuals of 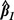 of GLS when the variance function of the error term is 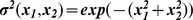.

**Figure 24:**
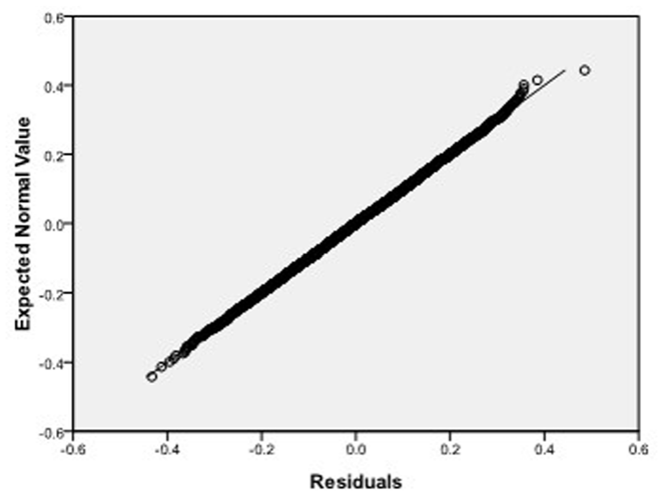
Normal Q-Q plot of residuals of 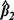 of GLS when the variance function of the error term is 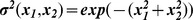.

**Figure 25:**
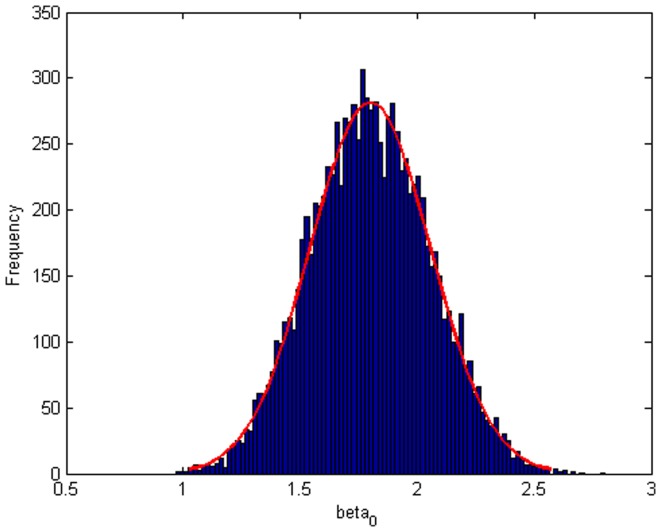
Histogram and asymptotic distribution for 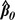 of OLS when the variance function of the error term is 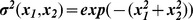.

**Figure 26:**
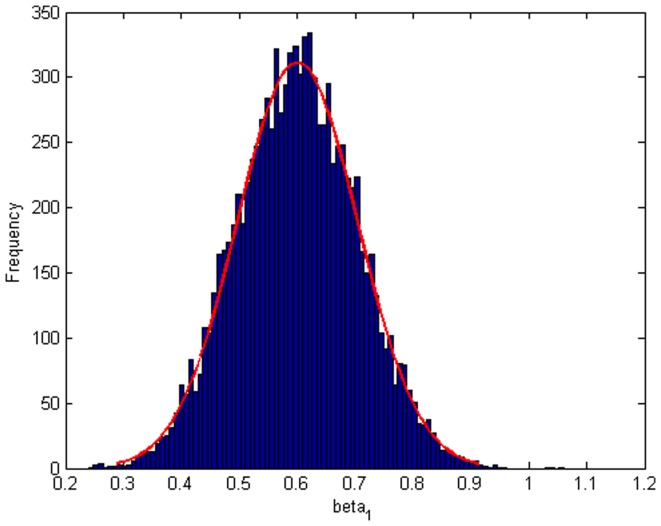
Histogram and asymptotic distribution for 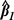 of OLS when the variance function of the error term is 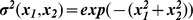.

**Figure 27:**
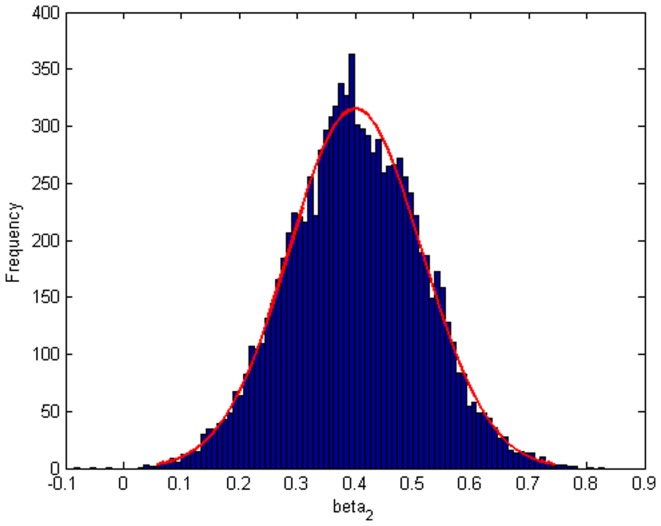
Histogram and asymptotic distribution for 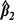 of OLS when the variance function of the error term is 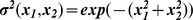.

**Figure 28:**
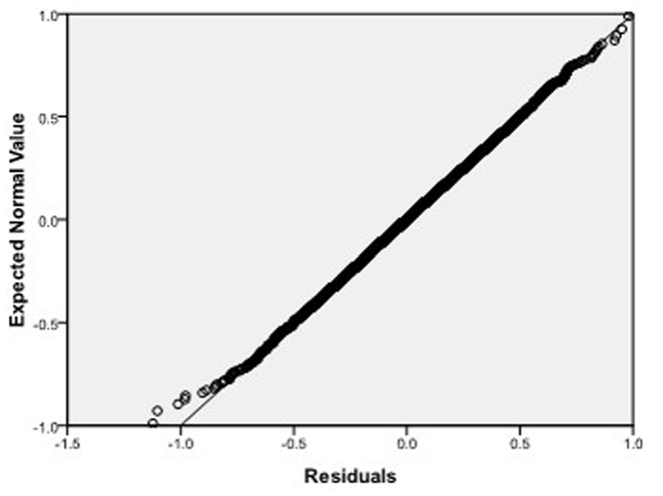
Normal Q-Q plot of residuals of 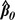 of OLS when the variance function of the error term is 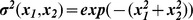.

**Figure 29:**
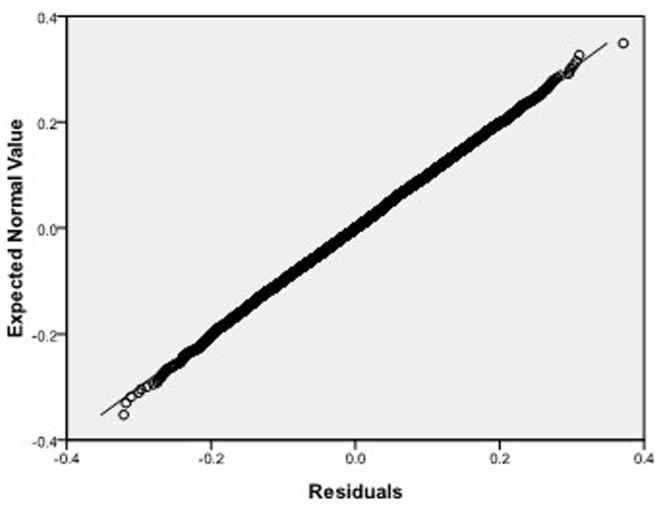
Normal Q-Q plot of residuals of 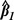 of OLS when the variance function of the error term is 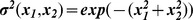.

**Figure 30:**
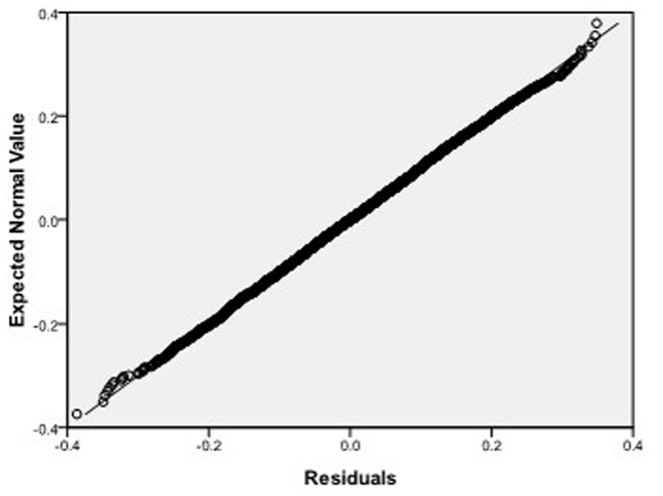
Normal Q-Q plot of residuals of 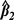 of OLS when the variance function of the error term is 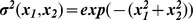.

Figure 18 indicates the plot of drawing fitted plot and original plot together. According to Figures 16, 17, 18, we can easily reach the conclusion that our fitting is nice and close to the true value. The histograms and asymptotic distributions of 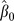, 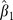 and 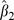 are depicted in Figures 19, 20 and 21. Besides, Figures 22, 23 and 24 contain the normal Q-Q plots of residuals, respectively. It is worth pointing out that the asymptotic distributions of parameters are symmetric. As shown in Figures 25, 26 and 27, the histograms and asymptotic distributions of 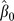, 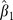, and 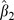 of OLS are not symmetrical, but irregular. There are larger fluctuations in normal Q-Q plots of residuals in Figures 28, 29 and 30 than those in Figures 22, 23 and 24. So it is not what we want to reach. Besides, we list fitted value and true value of 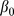, 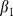, and 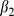 of GLS and ones of OLS in Table 3. In order to obtain a visual effect, we calculate the relative errors and do a comparison. It is easy to see that the GLS estimates of parameters are much better than OLS from Table 3.

**Table 3:** Two-stage estimates for 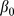, 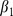, and 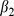 of GLS and OLS.

true value	1.8	0.6	0.4
GLS fitted value	1.79996	0.60004	0.39992
GLS relative error	0.0022%	0.0067%	0.02%
OLS fitted value	1.7992	0.6045	0.3917
OLS relative error	0.044%	0.75%	2.075%

### Application

As an example of pure cross-sectional data with potential for heteroscedasticity, consider the data given in Table 4, which gives data on per capita consumption expenditure (PCCE), agricultural business income (ABI) and other income (OI) for 31 different regions in China in 2009. The cross-sectional data presented in this table are quite heterogenous in a regression of PCCE on ABI and OI, so heteroscedasticity is likely.

**Table 4:** The data of PCCE, ABI and OI for regions in China in 2009.

areas	PCCE	ABI	OI
xinjiang	1350.23	1300.10	410.30
neimenggu	1554.60	1497.80	480.50
jilin	1661.70	1634.60	547.60
heilongjiang	1604.50	1684.10	596.20
xizang	1123.71	589.60	614.40
yunnan	1336.25	889.40	644.30
guizhou	1098.39	764.00	647.80
qinghai	1334.45	803.80	753.50
hainan	1357.43	1386.70	839.80
guangxi	1550.62	1068.80	875.60
shanxi	1331.03	614.80	876.00
gansu	1127.37	621.60	887.00
ningxia	1388.79	859.60	963.40
hubei	1649.20	1352.00	1000.10
anhui	1412.40	1013.10	1006.90
henan	1375.60	1083.80	1014.10
sichuan	1497.52	919.30	1067.60
chongqing	1475.16	883.20	1088.00
jiangxi	1720.00	1027.80	1203.80
liaoning	1786.30	1254.30	1303.60
shanxi	1221.60	609.80	1346.20
hunan	1990.30	908.20	1391.30
shandong	1905.00	1293.00	1511.60
hebei	1429.80	928.80	1674.80
fujian	2503.10	1053.00	2327.70
guangdong	2703.36	1242.90	2526.90
jiangsu	2374.70	1177.60	2607.20
tianjin	2050.90	1314.60	2633.10
zhejiang	3479.20	985.80	3596.60
beijing	3552.10	579.10	4446.40
shanghai	4753.20	652.50	5218.40

We hope to understand the relationship of PCCE, ABI and OI, then the regression function is as follows:



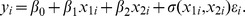
.

**Figure 31:**
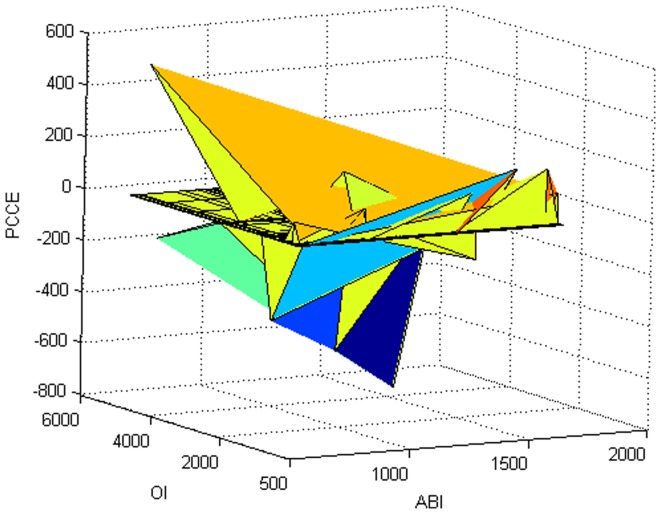
Variance squares.

Firstly, the ordinary least squares estimators can be obtained, say, 
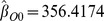
, 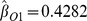, 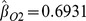. Then, inspect that if there is a heteroscedasticity. According to formula (23), the residual squares can be considered as variances. So we need only to fit residual squares, seeing Figure 31, where residual squares are variance squares. Obviously, there are biases among the residual squares fitted. Therefore, it can be concluded that there is a heteroscedasticity. Furthermore, according to the two-stage estimate, the generalized least squares estimation for 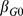, 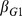, and 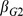 can be estimated after 10000 replicates provided with the bandwidth matrix 
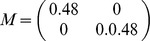
, the order 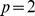, and 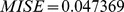. The estimations are 
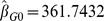
, 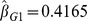, and 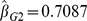. Finally, the regression equation of GLS can easily be written as follow:
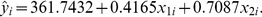
(30)


**Figure 32:**
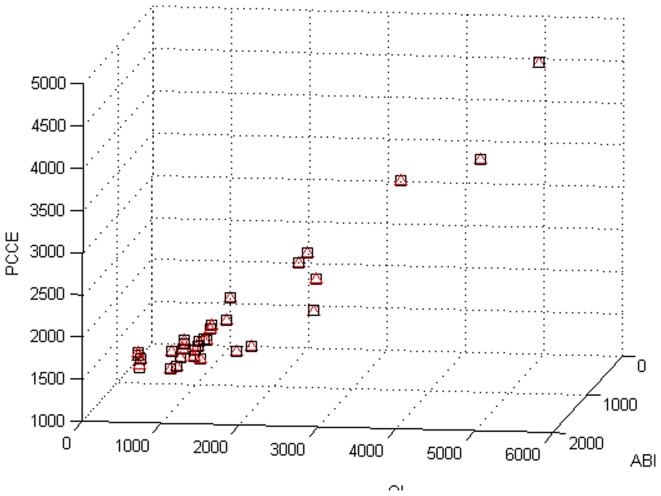
Scatter of original and fitting data.

The scatter of original and fitting data is also given, see Figure 32.

### Conclusions

In this paper £ we presented a new method for estimation of multivariate linear heteroscedastic regression model based on multivariate local polynomial estimation with non-parametric technique. The proposed scheme firstly adopted the local polynomial fitting to estimate heteroscedastic function, then the coefficients of regression model are obtained based on generalized least squares method. Our approach avoided the test of heteroscedasticity for the multivariate linear model. Due to non-parametric technique of local polynomial estimation, if the heteroscedastic function is unknown, the precision of estimation was improved. Furthermore, the asymptotic normality of parameters was verified by the results of numerical simulations normal Q-Q plots. Finally, the simulation results and local polynomial estimation of real data really indicated that our approach was effective in finite-sample situations, which did not need to assume the form of heteroscedastic function. The presented algorithm could be easily used to heteroscedastic regression model in some practical problems.
