# Correction: Local Polynomial Estimation of Heteroscedasticity in a Multivariate Linear Regression Model and Its Applications in Economics

**DOI:** 10.1371/annotation/01ebf0b5-ccd3-494d-b577-170981f7bc36

**Published:** 2012-11-20

**Authors:** Liyun Su, Yanyong Zhao, Tianshun Yan, Fenglan Li

There is an error in reference 21. The correct reference is: Takeda H, Farsiu S, and Milanfar P (2007) Kernel Regression for Image Processing and Reconstruction, IEEE Trans Image Process, 16( 2): 349-366. 

